# SiO_2_ Coated Up-Conversion Nanomaterial Doped with Ag Nanoparticles for Micro-CT Imaging

**DOI:** 10.3390/nano11123395

**Published:** 2021-12-15

**Authors:** Wei Zhang, Yanli Lu, Yang Zang, Jinhui Han, Qingyun Xiong, Jinping Xiong

**Affiliations:** 1 Beijing Key Laboratory of Electrochemical Process and Technology of Materials, Beijing University of Chemical Technology, Beijing 100029, China; 2019200387@mail.buct.edu.cn (W.Z.); 2019310036@mail.buct.du.cn (Y.L.); 2019310030@mail.buct.edu.cn (Y.Z.); 2020200494@mail.buct.edu.cn (Q.X.); 2 State Key Laboratory of Organic-Inorganic Composites, Beijing University of Chemical Technology, Beijing 100029, China; 2021400008@buct.edu.cn; 3 College of Ecology and Resources Engineering, Wuyi University, Wuyishan 354300, China

**Keywords:** up-conversion, nanomaterials, CT imaging

## Abstract

In this study, a new method for synthesizing Ag-NaYF_4_:Yb^3+^/Er^3+^ @ SiO_2_ nanocomposites was introduced. Using a hydrothermal method, the synthesized Yb^3+^- and Er^3+^-codoped NaYF_4_ up-conversion luminescent materials and Ag nanoparticles were doped into up-conversion nanomaterials and coated with SiO_2_ up-conversion nanomaterials. This material is known as Ag-UCNPs@SiO_2_, it improves both the luminous intensity because of the doped Ag nanoparticles and has low cytotoxicity because of the SiO_2_ coating. The morphology of UCNPs was observed using scanning electron microscopy (SEM), and the mapping confirmed the successful doping of Ag nanoparticles. Successful coating of SiO_2_ was confirmed using transmission electron microscopy (TEM). Fluorescence spectra were used to compare changes in luminescence intensity before and after doping Ag nanoparticles. The reason for the increase in luminescence intensity after doping with Ag nanoparticles was simulated using first-principles calculations. The cytotoxicity of Ag-UCNPs@SiO_2_ was tested via the cell counting kit-8 (CCK-8) method, and its imaging ability was characterized using the micro-CT method.

## 1. Introduction

Generally, up-conversion rare-earth nanomaterials are used in many fields because of their high fluorescence intensity. Recently, their application in biomedicine has been extensively studied. Many researchers have proposed that they can be used in biological imaging because they provide considerable advantage in the fight against major diseases such as cancer [1,2,3]. However, the common limitation is that up-conversion rare-earth nanomaterials have insufficient luminous intensity and are toxic to biological cells; therefore, the modification of structure and surface is necessary. To increase luminous intensity, many researchers proposed doping Mo^3+^, Cu^2+^, and other metal ions in the NaYF_4_: Yb^3+^/Er^3+^ unit cell; however, the effect is not significant [4,5,6]. Other studies proposed doping with Ag, which has a significant effect but Ag has a high light-to-heat conversion efficiency and can cause cell apoptosis without targeting; therefore, Ag cannot be used in biological studies [7,8]. Many researchers proposed developing core-shell structures such as NaYF_4_: Yb^3+^/Er^3+^@NaGdF_4_: Yb^3+^ and NaYF_4_: Yb^3+^/Er^3+^@NaNdF_4_: Yb^3+^/Tm^3+^@NaGdF_4_: Yb^3+^ [9,10,11]. Alternatively, the material surface is covered with a biocompatible coating such as DSPE-PEG_2K_ and ICG [12,13,14]. Although these operations can reduce biological toxicity and meet the basic requirements for use in biological cells or animals, these structures will indeed reduce the luminous intensity of up-conversion luminescent materials [15,16,17]. If such a material is used as a contrast agent for CT imaging, the image will be unclear; moreover, the tumor cannot be observed and additional diagnosis and treatment will be difficult [18,19,20]. Therefore, this study proposes a new structural up-conversion nanomaterial that has both extremely low cytotoxicity and good luminescence intensity and good targeting that can accurately label tumor cells and can be used for in-vivo imaging and obtain clear tumor images using Micro-CT. Many silver doped up-conversion rare earth materials have been studied before. Xu [21] investigated how the distance between silver and rare earth oxide in Ag-SiO_2_-Er_2_O_3_ changes the luminescence intensity of up conversion. Liu [22] and Xu did the same work, only changing the positions of silver nanoparticles and rare earth nanoparticles. Fan [23] on the basis of Xu, sensitizer Er is doped in rare earth oxide Y_2_O_3_ by high temperature eutectic. The study used a reverse microemulsion method to observe the negative outer layer of SiO_2_ shell on the silver nanoparticles or rare earth (RE) oxides, and then RE oxide or silver nanoparticles in the outer layer of SiO_2_. However, the relationship between the doping mode of silver nanoparticles, the photothermal conversion performance of silver nanoparticles and the up-conversion luminescence mechanism of rare earth has not been systematically studied. Therefore, their combination will often produce surprises. This study proposes using the coprecipitation-hydrothermal-reverse microemulsion (CHRm) method to synthesize the core-shell structure of Ag-UCNPs-SiO_2_. First, the copolymer of Ag nanoparticles and NaREF_4_ was obtained using a co-precipitation method. Secondly, this copolymer was transformed to Ag-UCNPs in a hexagonal crystal form using a hydrothermal method. Finally, a SiO_2_ layer was coated using a reverse microemulsion method.

## 2. Experimental

### 2.1. Materials

Y_2_O_3_ (99.99%), Yb_2_O_3_ (99.99%), Er_2_O_3_ (99.99%), nitric acid (68%), sodium fluoride (99.99%), citric acid (99.99%), cyclohexane (99.5%), sliver nitrate (99.99%), ethylenediaminetetraacetic acid (EDTA, ≥99%), sodium hydroxide (≥98%), and polyethylene pyrrolidone (PVP, average molecular weight of 1,000,000–1,500,000) were purchased from Aladdin (Aladdin, Los Angeles, CA, USA). The cell counting kit 8 (CCK-8) assay kit was purchased from BOVOGEN (BOVOGEN, Keilor East, Australia). All chemicals were used as received without additional purification.

### 2.2. Synthesis of Ag Nanoparticles

By dropping, 60 mL of 0.05 mol/L of citric acid solution was added to 3 mL of 0.02 mol/L of AgNO_3_ to obtain a mixture. Moreover, after 5 min of continuous stirring, the solution was transferred to a 100 mL reactor and placed in an oven for the reaction at 120 °C for 6 h. The reaction was then cooled to room temperature, washed, and centrifuged to obtain solid Ag nanoparticles, and then added to 10 mL of deionized water and PVP, and then placed in a test tube to prepare the sol for use.

### 2.3. Synthesis of Ag-UCNPs

RE_2_O_3_ (RE = Y, Yb, Er) was heated to achieve complete dissolution in excess nitric acid and then transferred to a vacuum system for evaporation to obtain a solid RE(NO_3_)_3_, which was then dissolved in deionized water and recrystallized twice. A certain amount of solid RE(NO_3_)_3_ (Y: Yb: Er = 0.79: 0.18: 0.03) was dissolved in deionized water, and EDTA (molar ratio of EDTA: RE(NO_3_)_3_ = 1:1) was added and stirred at 600 rpm for 1 h, the mixture was then weighed and dissolved in sodium fluoride in deionized water by ultrasound, and then the solution was added and stirred at 600 rpm for 1 h. Finally, the pH value was adjusted to 5.5 with NaOH, then 0 mL and 10 mL, respectively, of silver were added and the solution placed in a hydrothermal kettle to react at 190 °C for 24 h. The reaction products were cooled, centrifuged, and washed twice with ethanol/deionized water (1:1 *v*/*v*), and dried in vacuum at 80 °C for 3 h. The resultant powder is dispersed in cyclohexane for later use.

### 2.4. Synthesis of Ag-UCNPs@ SiO_2_

Note that 0.0083 g of Ag-UCNPs, 0.1 mL of CO-520 and 10 mL of cyclohexane were mixed and stirred for 10 min. Moreover, 0.4 mL of CO-520 and 0.08 mL of 30 wt% ammonia were added and the container was sealed and sonicated for 20 min until a transparent emulsion was formed. Furthermore, 0.04 mL of TEOS (variable concentration used determined by the distance between AgNPs and NaYF_4_: Yb, Er) was then added to the solution. The solution was stirred for 48 h at 1000 rpm. Finally, the product was collected by centrifugation, washed with deionized water and ethanol twice, and then re-dispersed in 6.0 mL of ethanol.

### 2.5. Characterization

Transmission electron microscopy (TEM) measurements were performed on a JEOL 2011 microscope operating at 200 kV. All samples were first dispersed in ethanol and then collected using a Cu grid covered with a carbon film for measurement. To determine the elemental composition of the samples, energy-dispersive X-ray spectroscopy (EDS) of the samples was performed on a JEOL 2010 EDS instrument using high-resolution transmission electron microscopy (HRTEM) measurements. Inductively coupled plasma-atomic emission spectrometry (ICPAES) was performed using a Perkin Elmer 7300DV apparatus. Scanning electron microscopy (SEM) images were obtained using a Philips XL30 electron microscope operating at 20 kV. Before this characterization, a Au film was sprayed on the sample. The up-conversion luminescence spectrum was obtained using a spectrum analyzer (ANDO AQ6317, Tokyo, Japan). The sample was placed in a 1.0 cm path length support, which was excited using a 980 nm CW semiconductor diode laser (Pmax 800 mW, 1000 mA). The up-conversion luminescence spectrum was obtained by the spectrophotometer using a multimode fiber having a core diameter of 0.6 mm. The distance between the top of the fiber and the sample is ~2 mm. 

### 2.6. CCK-8 Assay for Cytotoxicity

The culture medium in the flask was sucked out, washed with PBS, and then 0.25% of trypsin was added to digest cells after culturing HeLa cells in the logarithmic growth phase. After the removal of trypsin, the DMEM medium containing 10% fetal bovine serum was added to blow the cells, which were then transferred to the sampling tank and blown well. Subsequently, 100 µL cells were injected into a 96-well plate (1 × 10^4^ cells/well) and incubated for 24 h in a constant temperature incubator at 37 °C (5% CO_2_). The cells were incubated for 1.5 h in an incubator at 37 °C with 5% CO_2_ in accordance with concentrations of 200, 300, 400, 500, and 600 µg/mL. The culture medium was blotted out, PBS was rinsed twice, the culture medium was replaced in the 96-well plates with 100 µL of fresh DMEM containing 10% fetal bovine serum, and then 10 µL of CCK-8 solution was added to each well. The absorbance of each well at 450 nm was measured using a microplate reader after 2 h of culturing in the incubator. The cell survival rate calculation formula is as follows:

Cell survival rate (%) = (A sample)/(A control) × 100%

## 3. Results and Discussion

**Figure 1:**
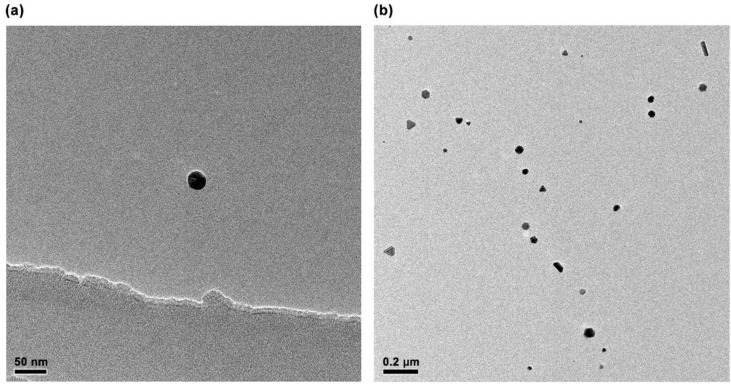
TEM image of Ag nanoparticles (**a**): the scale of 50 nm, (**b**): the scale of 0.2 µm.

**Figure 2:**
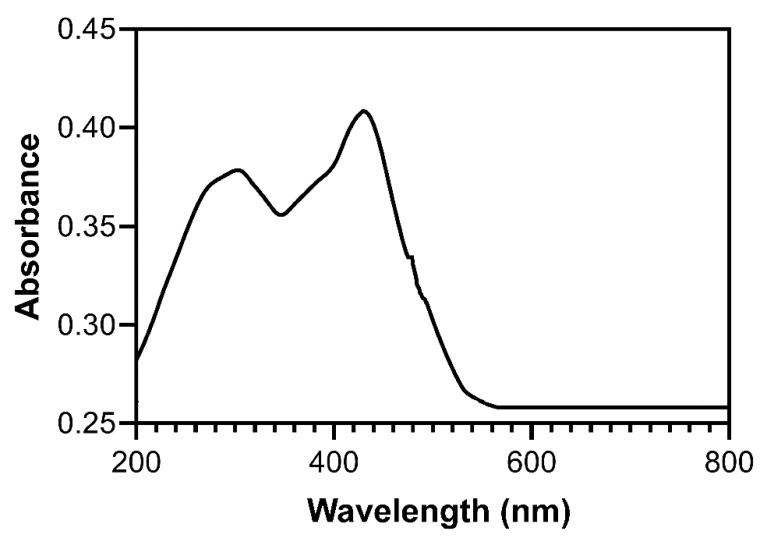
UV absorption spectrum of Ag nanoparticles.

The TEM images of Ag nanoparticles (Figure 1) prepared using the hydrothermal method show that they are spherical and have an average diameter of ~30 nm. The UV-visible absorption spectra of Ag nanoparticles (Figure 2) obtained using a UV absorption spectrophotometer shows that the absorption peak of Ag nanoparticles is located at 430 nm, which is consistent with the results reported in the literature.

**Figure 3:**
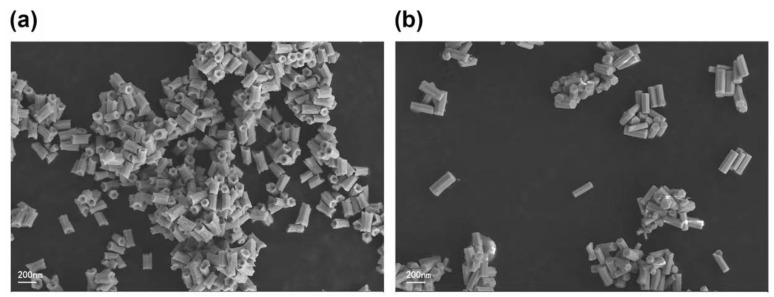
SEM image of UCNPs (**a**) and Ag-UCNPs (**b**).

**Figure 4:**
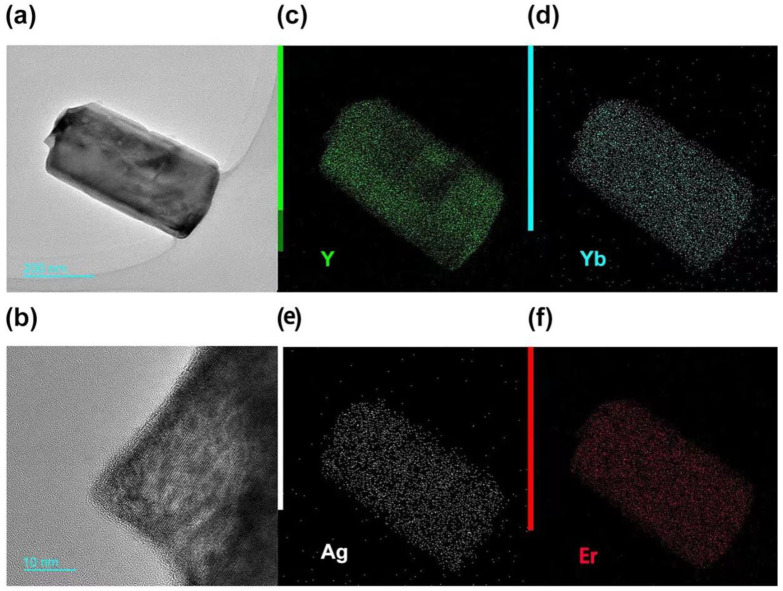
(**a**): TEM image of Ag-UCNPs, (**b**): Lattice of Ag-UCNPs, (**c**–**f**): mapping of (**a**).

The SEM image shows the normal hexagonal crystal of UCNPs (Figure 3a) has defects on both ends and sides, whereas the hexagonal crystal of Ag-UCNPs (Figure 3b) doped with Ag is smooth. This smooth crystal structure then increases luminous intensity, and this view was confirmed by comparing their luminous intensities. The elemental distribution characterized by mapping confirms the successful doping of Ag nanoparticles (Figure 4).

**Figure 5:**
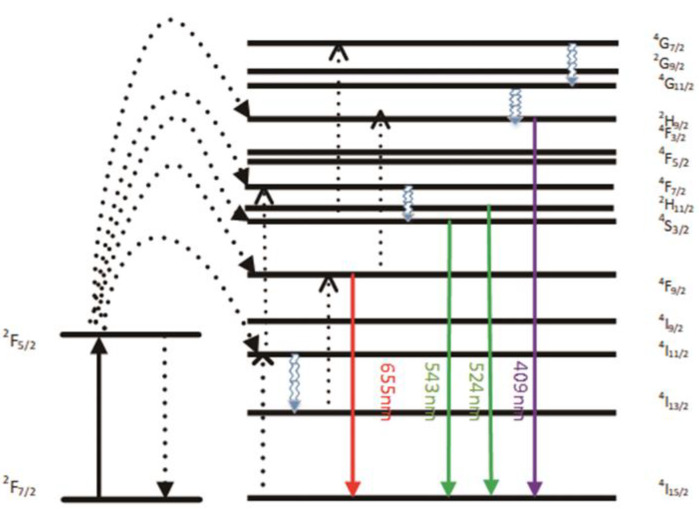
A simplified energy level diagram of Er^3+^/Yb^3+^ system embedded with AgNPs and possible up-conversion pathways.

**Figure 6:**
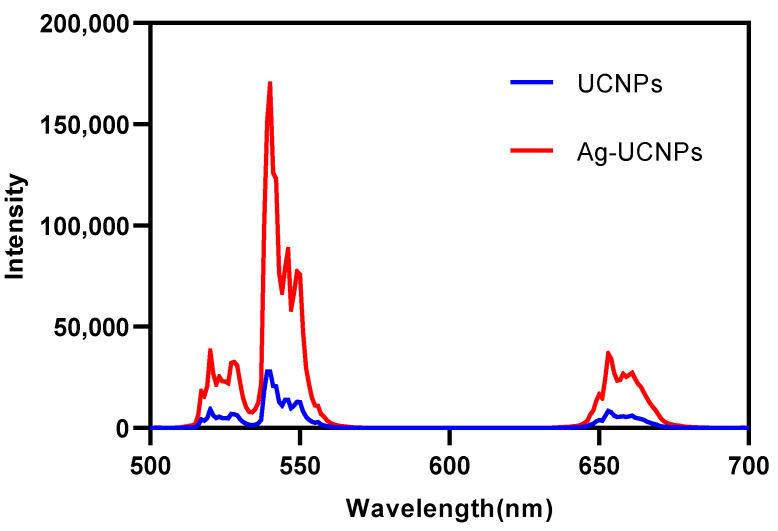
Luminescent intensity of UCNPs and Ag-UCNPs.

Figure 5 and Figure 6 show that the emission bands are observed at 409 nm(purple), 524 nm, 543 nm (green), and 655 nm (red) are due to the ^2^H_9/2_ → ^4^I1_5/2_, ^2^H_11/2_ → ^4^I_15/2_, ^4^S_3/2_ → ^4^I_15/2_ and ^4^F_9/2_ → ^4^I_15/2_ transitions of Er^3+^ ions, respectively. However, it is found that the red and green emission becomes more dominant than the purple emission, which may be due to the cross-relaxation process from ^2^G_7/2_ to ^2^H_9/2_ levels. In addition, purple light, green light, and red light show different degrees of enhancement. The SPR absorption peak at 520 nm coincides with the emission band of green light, so the SPR vibration frequency overlaps the luminescence band of UCNPs, the coupling of the emitted light and the SPR will increase the photon localized state density near the surface of AgNPs, thereby increasing the radiation decay rate of Er^3+^, increasing the luminous intensity. Furthermore, the SPR effect of AgNPs produces a local electric field enhancement effect, which enhances the absorption of sensitizers through electric field coupling and also increases the emission intensity of UCNPs. Moreover, the excitation wavelength of 980 nm excites the higher lying ^4^F_7/2_ level of Er^3+^ ions and AgNPs partially absorb emissions coming from Er^3+^ ions, leading to de-excitation of the fluorescence of UCNPs. However, this part of the energy transferred to the surface of AgNPs will be radiated to the far field with a higher efficiency, which will also increase the fluorescence of UCNPs. The enhancement of the up-conversion luminescence helps to effectively mark the location of the drug.

Both UCNPs and Ag-UCNPs were prepared in 0.2 M solutions and their luminescence intensity (Figure 6) at a wavelength of 980 nm was tested. The results demonstrated that the luminescence intensity increased by ~6.5 times after doping with Ag. This phenomenon can be explained using first-principles calculations.

**Figure 7:**
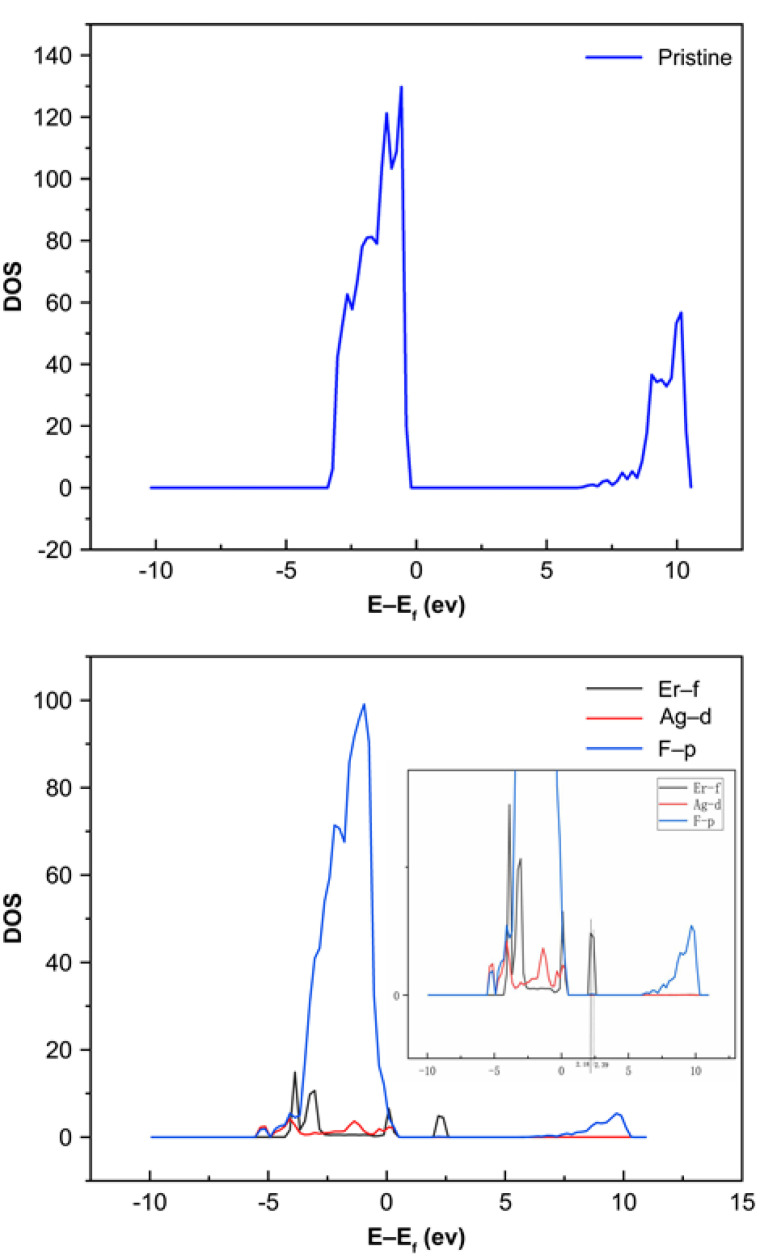
Computed DOS of the perfect NaYF_4_ and Er-Ag-NaYF_4_ doping structures and local enlarged image of Er-Ag-NaYF_4_ doping structures.

DFT calculations were performed to understand the electronic properties and chemical origins of Ag nanoparticles better. We selected Ag-NaYF_4_ to examine the effect of Ag nanoparticles on the Er luminescence center of materials because of the difficulty of convergence in multi-rare earth-doping systems. As shown in Figure 6, the calculated total density of states (DOS) shows a considerable bandgap for pure NaYF_4_. The bandgap calculated is 6.78 eV, which has an underestimated materials bandgap compared to experimental values. However, this is very common with DFT calculations, which are known to underestimate materials bandgap due to the insufficient processing of exchange association items. The bandgap then narrows and additional hybridized electronic states occur close to the Fermi level in Ag-NaYF_4_ when Ag nanoparticles are doped in Er-NaYF_4_, (Figure 7). As can be clearly seen in Figure 7, there exists a peak related to Er-f at 2.18 eV and 2.39 eV above the Fermi level, which is within the range of the photon energy of green glow. The hybridized electronic states close to the Fermi level are shown to be formed by the 4f orbital of Er’s 4d orbital of Ag and 2p orbital of F. The enhancement of total DOS close to the Fermi level in Ag-NaYF_4_ may make it easier to absorb photons and enhance luminescence.

**Figure 8:**
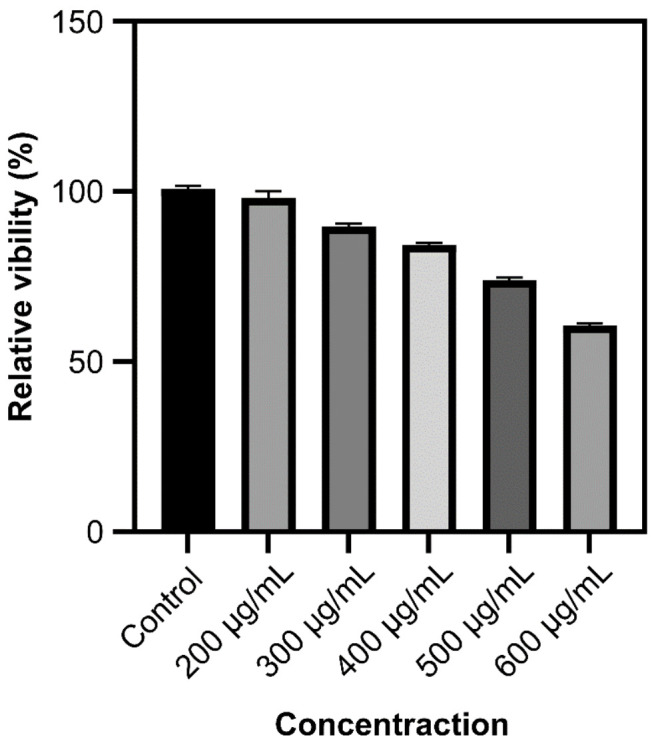
Cytotoxicity of different concentrations of Ag-UCNPs.

Although this luminescence intensity is sufficient for imaging organisms, it does not solve the biotoxicity problem (Figure 8).

**Figure 9:**
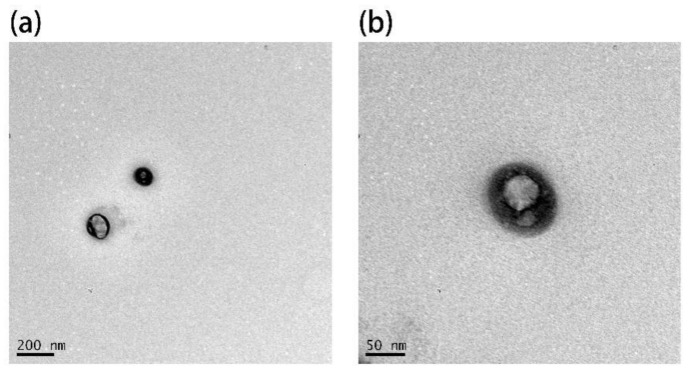
TEM image of Ag-UCNPs@SiO_2_, (**a**): the scale of 200 nm, (**b**): the scale of 50 nm.

A reverse microemulsion method was used to fabricate a SiO_2_ shell structure coated with Ag-UCNPs. Moreover, TEM was used to confirm that SiO_2_ successfully coated Ag-UCNPs (Figure 9).

**Figure 10:**
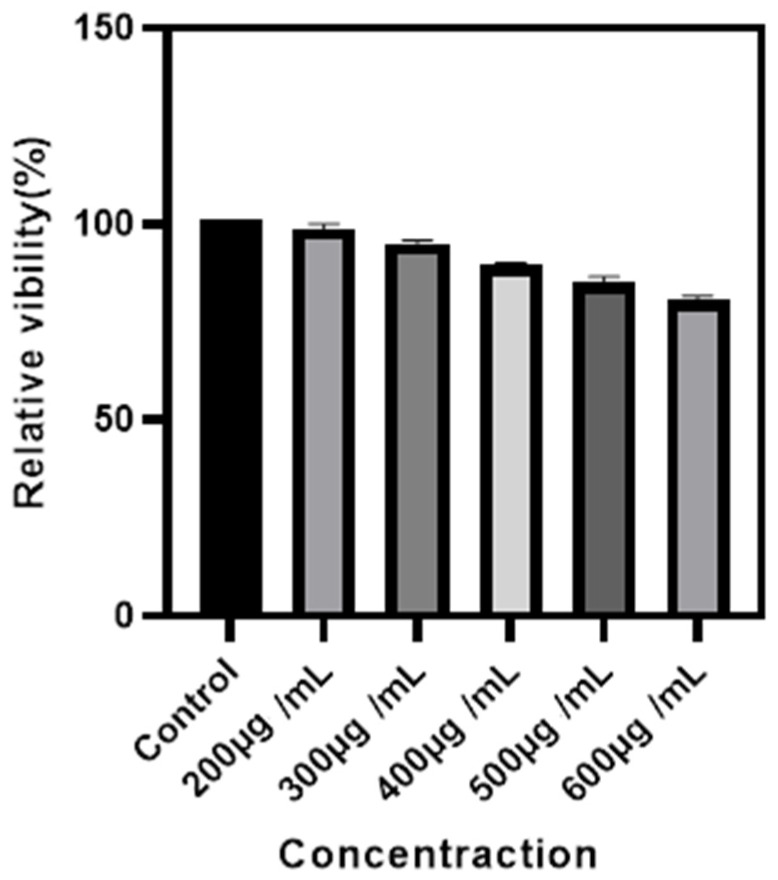
Cytotoxicity of different concentrations of Ag-UCNPs@SiO_2_.

Modified rare-earth nanomaterials were dispersed in normal saline to prepare different concentrations after which HeLa cells were cultured for 4 h and their activity was tested (Figure 10). When the concentration is <400 µg/mL, the cell survival rate is >89%. In particular, at 200 µg/mL, the cell survival rate was >99%. Combined with Figure 5, the luminescence intensity of rare earth, 200 µg/mL concentration of rare-earth nanomaterials not only have sufficient safety but also have a high luminous intensity. When the rare-earth ion concentration is as high as 500 or even 600 µg/mL, the cell survival rate is still >80%.

**Figure 11:**
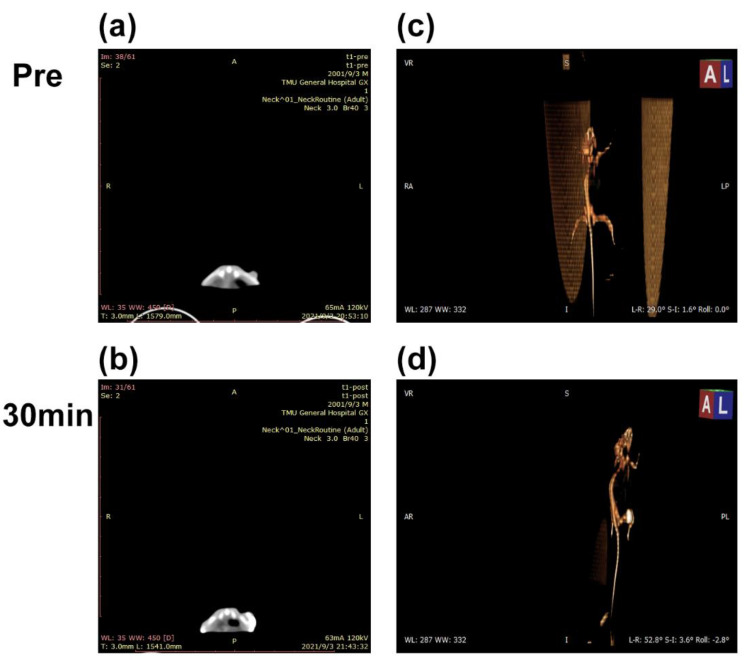
Micro-CT images before (**a**) and after (**b**) intratumor injection of Ag-UCNPs@SiO_2_ in Balb/c mice, (**c**) and (**d**) are the 3-D render of (**a**) and (**b**).

A 200 µg/mL concentration of Ag-UCNPs@SiO_2_ was intratumorally injected in the mice. Figure 11c shows that the tumor site of the mouse before injection has no signal under Micro-CT imaging; furthermore, 30 min after the injection of Ag-UCNPs-SiO_2_ (Figure 11d), the tumor site of the mouse shows a CT signal. The results firmly demonstrate that the Ag-UCNPs@SiO_2_ effectively serves as a contrast agent for CT imaging in vivo.

**Figure 12:**
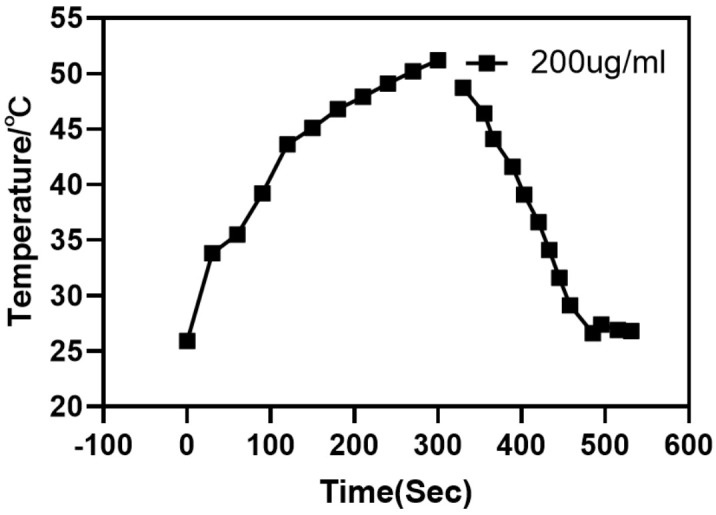
980 nm wavelength of near-infrared light excites the temperature rising-falling curve of 200 µg/mL of Ag-UCNPs@SiO_2_.

Compared with the contrast ability of Ag-UCNPs@SiO_2_, its particularity lies in its photothermal conversion efficiency (Figure 12). It is excited by near-infrared light at 980 nm wavelength, it is observed by thermal imager to not only emit green fluorescence (Figure 6), but also emit heat. Combined with its biocompatibility, Ag-UCNPs@SiO_2_ can be considered as photothermal therapy reagent. The reason for this phenomenon is that silver will generate heat when irradiated by 540 nm laser [24], while rare earth up-conversion nano materials excited by 980 nm near-infrared light will emit 540 nm fluorescence (Figure 6). Secondly, the doping of silver nanoparticles will enhance the energy of 540 nm wavelength (Figure 7). Finally, the energy of 540 nm wavelength excites the silver nanoparticles, making the silver nanoparticles release heat. Incidentally, the carrier properties of 540 nm laser, 980 nm near-infrared light and 540 nm fluorescence are the same, and the photothermal results also show that.

## 4. Conclusions

The doped Ag nanoparticles increase the luminous intensity of UCNPs, and SiO_2_, which has excellent biocompatibility, was used as the shell material; it greatly improves the luminous intensity of up-conversion nano-materials and has extremely low cytotoxicity. These nanoparticles can be used as an excellent biomedical material. Second, compared with the traditional medical imaging, tumor images can be observed in the in-vivo single-mode imaging system. The images can play an important role in future multi-mode imaging systems, thus providing complete and clear images for diagnosing and treating complex cancers such as blood metastasis. Finally, the extremely high luminous intensity of this material is primarily attributed to Ag nanoparticles, which have good photothermal conversion efficiency. In future, this material could be used along with photothermal therapy (PTT) and multi-mode imaging, which is used to complete the cancer treatment and diagnosis.

## Data Availability

Not applicable.
